# The clinical and therapeutic profiles of prolactinomas associated with germline pathogenic variants in the *aryl hydrocarbon receptor interacting protein* (AIP) gene

**DOI:** 10.3389/fendo.2023.1242588

**Published:** 2023-08-29

**Authors:** Laurent Vroonen, Albert Beckers, Severine Camby, Thomas Cuny, Pablo Beckers, Marie-Lise Jaffrain-Rea, Muriel Cogne, Luciana Naves, Amandine Ferriere, Pauline Romanet, Atanaska Elenkova, Auli Karhu, Thierry Brue, Anne Barlier, Patrick Pétrossians, Adrian F. Daly

**Affiliations:** ^1^ Department of Endocrinology, Centre Hospitalier Universitaire de Liège, University of Liège, Liège, Belgium; ^2^ Department of Otorhinolaryngology, Centre Hospitalier Universitaire de Liège, University of Liège, Liège, Belgium; ^3^ Department of Endocrinology, Aix Marseille University, Assistance publique Hôpitaux de Marseille (APHM), INSERM, Marseille Medical Genetics (MMG), Hopital La Conception, Institut MarMaRa, Marseille, France; ^4^ Department of Human Genetics, Centre Hospitalier Universitaire de Liège, University of Liège, Liège, Belgium; ^5^ Department of Biotechnological and Applied Clinical Sciences, University of L’Aquila, L’Aquila, Italy; ^6^ Department of Neuroendocrinology, Neuromed IRCCS, Pozzilli, Italy; ^7^ Department of Endocrinology, Diabetes and Nutrition, Centre Hospitalo-Universitaire de la Réunion, Saint-Pierre, France; ^8^ Department of Endocrinology, University of Brasilia, Brasilia, Brazil; ^9^ Department of Endocrinology, Hopital Haut-Leveque, Centre Hospitalier Universitaire (CHU) de Bordeaux, Pessac, France; ^10^ Laboratory of Molecular Biology, Aix Marseille University, Assistance publique Hôpitaux de Marseille (APHM), INSERM, Marseille Medical Genetics (MMG), Hospital La Conception, Institut MarMaRa, Marseille, France; ^11^ Department of Endocrinology, Medical University of Sofia, Sofia, Bulgaria; ^12^ Department of Medical and Clinical Genetics, University of Helsinki, Helsinki, Finland; ^13^ Applied Tumor Genomics Research Program, Research Programs Unit, University of Helsinki, Helsinki, Finland

**Keywords:** prolactinoma, genetic, resistance, dopamine agonist, cabergoline, AIP, MEN1

## Abstract

**Introduction:**

Prolactinomas are the most frequent type of pituitary adenoma encountered in clinical practice. Dopamine agonists (DA) like cabergoline typically provide sign/ symptom control, normalize prolactin levels and decrease tumor size in most patients. DA-resistant prolactinomas are infrequent and can occur in association with some genetic causes like MEN1 and pathogenic germline variants in the *AIP* gene (AIPvar).

**Methods:**

We compared the clinical, radiological, and therapeutic characteristics of AIPvar-related prolactinomas (n=13) with unselected hospital-treated prolactinomas (“unselected”, n=41) and genetically-negative, DA-resistant prolactinomas (DA-resistant, n=39).

**Results:**

AIPvar-related prolactinomas occurred at a significantly younger age than the unselected or DA-resistant prolactinomas (p<0.01). Males were more common in the AIPvar (75.0%) and DA- resistant (49.7%) versus unselected prolactinomas (9.8%; p<0.001). AIPvar prolactinomas exhibited significantly more frequent invasion than the other groups (p<0.001) and exhibited a trend to larger tumor diameter. The DA-resistant group had significantly higher prolactin levels at diagnosis than the AIPvar group (p<0.001). Maximum DA doses were significantly higher in the AIPvar and DA-resistant groups versus unselected. DA-induced macroadenoma shrinkage (>50%) occurred in 58.3% in the AIPvar group versus 4.2% in the DA-resistant group (p<0.01). Surgery was more frequent in the AIPvar and DA- resistant groups (43.8% and 61.5%, respectively) versus unselected (19.5%: p<0.01). Radiotherapy was used only in AIPvar (18.8%) and DA-resistant (25.6%) groups.

**Discussion:**

AIPvar confer an aggressive phenotype in prolactinomas, with invasive tumors occurring at a younger age. These characteristics can help differentiate rare AIPvar related prolactinomas from DA-resistant, genetically-negative tumors.

## 1 Introduction

Prolactinomas are the most frequent (53%) clinically relevant pituitary adenomas, occurring with a prevalence of about 1 case in every 2000 of the general population (1). Typically, prolactinomas are diagnosed in premenopausal women, most often as microadenomas that present with menstrual disturbance and galactorrhea (2). With increasing age, the epidemiology of prolactinomas changes, with males accounting for an increasing proportion of patients (3, 4). In general, dopamine agonists (DA), such as, cabergoline are the first-line treatment for prolactinomas, as these agents are effective in normalizing prolactin secretion and controlling signs and symptoms in the majority of cases. Prolactinomas that are resistant to DA at labelled doses may require careful dose escalation, or referral for neurosurgical resection (5, 6).

Despite being the predominant form of pituitary adenomas, relatively little is known about the pathophysiology of prolactinomas. Most pituitary adenomas are sporadic and are believed to arise from somatic genetic changes in a single cell that later undergoes clonal expansion (7). Few consistent molecular genetic abnormalities have been identified to date in studies of prolactinoma tissue. Prolactinomas can occur as part of an inherited multiorgan endocrine tumor syndrome, such as multiple endocrine neoplasia (MEN) 1, MEN4, and pheochromocytoma/paraganglioma/pituitary adenoma (3P) Association (8). In MEN1, like in sporadic tumors, prolactinomas are the most frequent pituitary adenoma seen (9–11). Prolactinomas also occur in the setting of familial isolated pituitary adenoma (FIPA) kindreds, in which two or more related members in a family have isolated pituitary adenomas in the absence of MEN1 or other multiple endocrine neoplasias (12). In FIPA, prolactinomas tend to be larger and occur at a younger age than sporadic tumors (12). About 15-25% of FIPA kindreds have an underlying pathogenic *AIP* gene variant (*AIPvar*), which typically leads to acromegaly-gigantism, although many adenomas are mixed growth hormone and prolactin-secreting, but prolactinomas are seen occasionally (13–15). Apparently sporadic prolactinomas with an aggressive phenotype occurring in young individuals can also be due to *AIPvar* (16, 17). In acromegaly, *AIPvar* leads to a clinical phenotype of resistance to the first-generation somatostatin analogs, octreotide and lanreotide (16). Due to their comparative rarity, less is known about the characteristics of prolactinomas due to *AIPvar*, although DA resistance has been described (16). To better characterize the profile of patients with prolactinomas due to *AIPvar*, we performed a retrospective study in which the demographic, tumoral and therapeutic characteristics of *AIPvar* patients were compared with two control groups: an unselected group of prolactinomas from a real-world clinical setting and *AIP*-negative, DA-resistant prolactinoma patients.

## 2 Methods

This was an international, retrospective study performed in subjects with prolactinoma and an accompanying pathogenic or probably pathogenic germline variant in the *AIP* gene. The study population consisted of three groups: the *AIPvar* group, a dopamine agonist (DA)-resistant group, and an unselected prolactinoma group. The international, DA-resistant comparator group consisted of a series of subjects referred for genetic testing due to prolactinomas that were resistant to DA treatment at the maximum labelled dose and in which no germline variants or deletions in *AIP*, *MEN1*, or *CDKN1B* were found on genetic testing. In practice, DA resistance was taken as a lack of normalization of serum prolactin following treatment with the maximum labelled dose of 2 mg/week of cabergoline for at least 6 months (18). The second comparator group were subjects with prolactinomas that were not filtered or pre-selected in terms of clinical characteristics or genetic screening results. This group represented a “real-world” population studied at the Department of Endocrinology, Centre Hospitalier Universitaire de Liège, Belgium.

To be eligible, all subjects from all groups had to have proven radiological evidence of a pituitary adenoma and to have demonstrated elevated levels of prolactin that was associated with clinical symptoms. Individuals with non-tumoral, drug-induced or unexplained hyperprolactinemia were not included. The demographic and clinical characteristics of all subjects were collected and included the following criteria: age, sex, age at first symptoms and diagnosis, tumoral maximum diameter (mm) on magnetic resonance imaging (MRI), invasion (yes/no; unilateral, bilateral), extrasellar extension, optic chiasma compression, treatments used, surgery (number of interventions), radiotherapy, prolactin level at diagnosis and nadir levels under treatments.

Genetic testing for sequence variants in *AIP*, *MEN1* and *CDKN1B* was performed as previously described (13, 19). Only pathogenic and probably pathogenic variants were selected. In addition, multiplex ligation-specific probe amplification (MLPA) kits were used to identify deletions of whole or part of these three genes (SALSA^®^ MLPA^®^ Probemix P244, MRC-Holland, The Netherlands). Previously we reported the clinical characteristics of some of these pathogenic *AIPvar* patients but without comparisons with control groups (16). The study was approved by the Ethics Committee of the University of Liège and all patients provided informed consent.

### 2.1 Statistical analysis

Data on discrete variables were expressed as medians and interquartile ranges (Q1-Q3) and analyzed using Wilcoxon’s test. Categorical variables in the different groups were assessed using Pearson’s chi-squared test with Yates’ continuity correction. Statistical analyses were performed with the R software package (R Core Team 2015; http://www.R-project.org). Graphics were plotted using the ggplot2 library (https://ggplot2.tidyverse.org). *Ggplot2: Elegant Graphics for Data Analysis*. Springer-Verlag New York. ISBN 978-3-319-24277-4)

## 3 Results

The study population consisted of 13 subjects with *AIPvar*, 39 subjects with genetically-negative, DA-resistant prolactinomas and 41 unselected subjects with prolactinomas. There were 11 different germline *AIPvar* in the *AIPvar* group, all of which were previously described. Three variants in five subjects led to protein truncation: p.Gln14X (n=2), p.Tyr268X, and p.Gly117Alafs*39 (n=2). The remaining *AIPvar* were missense (p.Arg56Cys, p.Leu58Asn, p.Leu70Met, pVal195Ala, p.Lys241Glu, p.Tyr268Cys, and p.Arg271Trp) or a splice site change (c.100-18 C>T (IVS1)) in one subject each. Another three subjects with the variants p.Arg16His (n=1) and p.Arg304Gln (n=2), for which pathogenicity is debatable, were not included in the statistical analyses. Ten subjects came from heterogeneous acromegaly-prolactinoma FIPA kindreds (all subjects presented spontaneously and none had been identified on family screening) and three were apparently sporadic (without full available studies of parents’ *AIP* status).

**Figure 1:**
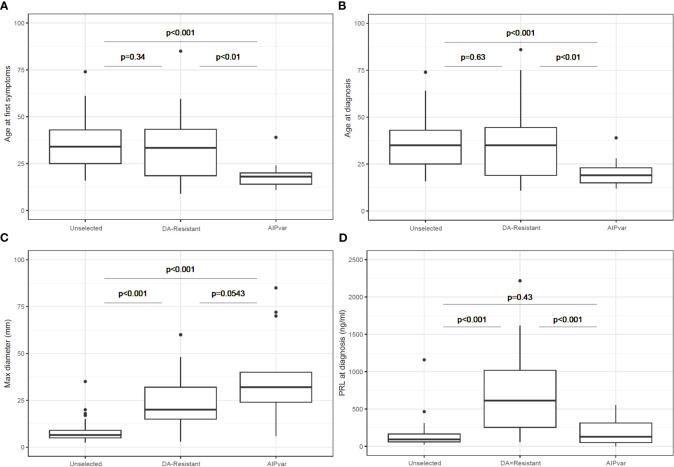
Comparisons between groups with *AIPvar-*related, unselected and DA-resistant prolactinomas in terms of age at first symptoms **(A)**, age at diagnosis **(B)**, maximum tumor diameter at diagnosis **(C)** and prolactin secretion at diagnosis **(D)**. Box and whisker plots show medians as horizontal lines and the lower and upper limits of the box correspond to the first and third quartiles; the whiskers extend the box to 1.5 times the inter-quartile range (IQR) or to the most extreme values if they lie within this range.

The sex distribution was significantly different between the unselected controls (female: 90.2%) and the *AIPvar* (female: 30.8%; p<0.001) and DA-resistant groups (female: 51.3%; p<0.001). There was no statistically significant difference between the *AIPvar* and DA-resistant groups in terms of sex distribution (p=0.14). The *AIPvar* group had a significantly younger median age at first symptoms (18.0 years; Q1-Q3: 15.0-23.0) than either the unselected prolactinoma (34.0 years; Q1-Q3: 25.0-43.0; p<0.001) or DA-resistant groups (33.0 years; 19.0-43.0; p<0.01, **Figure 1A**). Similarly, the median age at diagnosis was significantly younger in the *AIPvar* prolactinoma group (19.0 years; Q1-Q3:15.0-24.0) versus the unselected (35.0 years; Q1-Q3: 25.0-43.0; p<0.001) and DA-resistant groups (35.0 years; Q1-Q3: 19.0-44.0: p<0.01, **Figure 1B**).

*AIPvar*-associated prolactinomas had a significantly larger median maximum diameter (32.0 mm; Q1-Q3: 26-41) than the unselected prolactinoma group (6.5 mm; Q1-Q3: 5.0-9.0; p<0.001); there was a trend towards a larger tumor size in the *AIPvar* versus the DA-resistant groups (20.0 mm; Q1-Q3: 15.0-32.0; p=0.054: **Figure 1C**). At diagnosis, macroadenomas predominated in the *AIPvar* (92.3%) and DA-resistant groups (89.7%), whereas they constituted only 24.4% of the unselected prolactinomas. Giant adenomas (≥40 mm) were present in 5/13 (38.5%) of the *AIPvar* group, 7/39 (17.9%) of the DA-resistant and in none of the unselected patients (p<0.01 unselected vs *AIPvar*). Eleven of 13 (84.6%) *AIPvar* prolactinomas had suprasellar extension, versus 28.2% of the DA-resistant and 7.3% of the unselected groups (p ≤ 0.01 *AIPvar* vs DA-resistant or unselected). Also, chiasmal compression was >4 times more frequent at diagnosis in the *AIPvar* group (61.5%) as compared to the DA-resistant prolactinomas (12.8%) and >8-fold higher than in the unselected prolactinoma controls (p ≤ 0.01 *AIPvar* vs DA-resistant or unselected). Fully 76.9% of *AIPvar*-related prolactinomas had invaded the cavernous sinuses (30.8% bilaterally) at the time of diagnosis, as compared with 28.2% of DA-resistant prolactinomas (0% bilateral) and only 14.6% (0% bilateral) of the unselected group (p<0.001 *AIPvar* vs DA-resistant or unselected).

**Figure 2:**
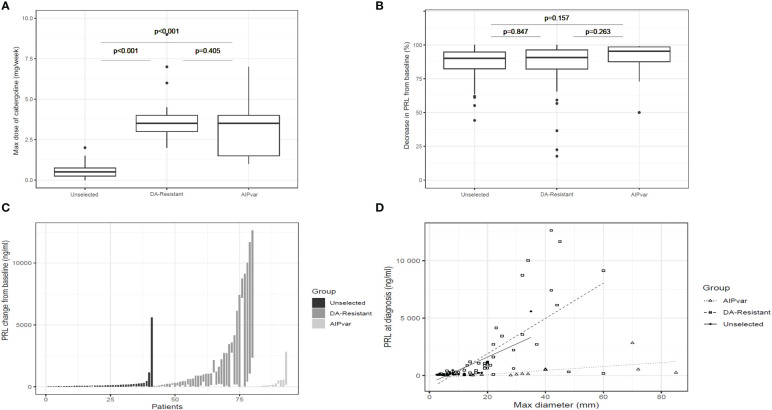
Dopamine agonist treatment of prolactinomas. Comparisons between groups with *AIPvar-*related, unselected and DA-resistant prolactinomas according to median weekly dose of cabergoline **(A)**; median percentage reduction from baseline at maximum cabergoline dose in the three study groups **(B)**; a waterfall plot depicts the individual reductions in prolactin from baseline to nadir levels under maximum cabergoline doses in the three groups **(C)**. The relationship of prolactinoma maximum diameter and prolactin secretion at baseline across the three study groups was compared in panel **(D)** Box and whisker plots show medians as horizontal lines and the lower and upper limits of the box correspond to the first and third quartiles; the whiskers extend the box to 1.5 times the inter-quartile range (IQR) or to the most extreme values if they lie within this range.

In contrast to the above differences in tumor characteristics, the DA-resistant prolactinoma group had a significantly higher prolactin level at diagnosis as compared with both the unselected controls and the *AIPvar* group (**Figure 1D**; p<0.001). There was no significant difference in prolactin levels at diagnosis between the *AIPvar* and unselected controls (p=0.43). There was no evidence of interference by elevated macroprolactin forms in the biochemical analyses of the *AIPvar* group. The median maximal dose of cabergoline in the unselected prolactinoma group was significantly lower (0.5: Q1-Q3: 0.25-0.75 mg) as compared with the *AIPvar* (3.5: Q1-Q3: 2.0-5.0 mg; p<0.001) and the DA-resistant groups (3.5: Q1-Q3: 3.0-4.0 mg; p<0.001, **Figure 2A**); *AIPvar* and DA-resistant groups did not differ significantly (p=0.41). The median decrease in prolactin from baseline at last follow-up was approximately the same across all three study groups (90.0-95.4%; **Figure 2B**). As shown in the waterfall plot in **Figure 2C**, however, the high prolactin at baseline in the DA-resistant group meant that these >90% decreases were insufficient to normalise prolactin levels in many cases. Hyperprolactinemia remained at the maximum dose of DA in 4/13 (30.7%) in the *AIPvar* group, 5/41 (12.2%) in the unselected group and in 26/39 (66.7%) of the DA-resistant group. We also examined the relationship between prolactin secretion at diagnosis and tumor diameter. As shown in **Figure 2D**, most tumors in the unselected group were small and the prolactin level steadily increased with rising tumor size ( ± 23.5 mg/dL per each mm of tumor diameter, p<0.001). In the DA-resistant group, tumors were larger and secreted more prolactin; this secretion rose significantly at a rate of ± 154.0 mg/dL for each mm increase in diameter (p<0.001). Prolactinomas in the *AIPvar* group, which were larger than in the unselected group, had a different secretory pattern, with the prolactin levels barely increasing by 5.8 mg/dL for each mm of tumor diameter (p=0.028).

Shrinkage of >50% following DA treatment occurred in 7/12 with evaluable MRI data in the *AIPvar* group, as compared with only 1/24 in the DA-resistant group (p<0.01). Surgery was more frequent in the *AIPvar* and DA-resistant groups (53.8% and 61.5%, respectively) as compared with the unselected prolactinomas (19.5%: p<0.01 for *AIPvar* and DA-resistant versus unselected patients). No patient in the unselected prolactinoma group received radiotherapy, whereas it was used in 23.1% and 25.6% of the *AIPvar* and DA-resistant groups, respectively (p<0.03 for *AIPvar* and DA-resistant versus unselected prolactinomas).

## 4 Discussion

Prolactinomas are the most frequently encountered pituitary adenoma in clinical practice (1, 20). In children, adolescents and young adults, prolactinomas are also the most common pituitary adenoma (21). Most present as microadenomas in fertile women that are responsive to labelled doses of cabergoline (2). Prolactinomas that are resistant or refractory to these DA doses are rarer, and some can have aggressive clinical characteristics (22, 23). The pathophysiology of DA resistance in prolactinomas is largely unknown, but factors like altered expression of dopamine D2 receptor levels have been suggested (24). Although AIP has been implicated in resistance to somatostatin analogs in *AIPvar* somatotropinomas (16, 25), this has not been explored in details in *AIPvar* prolactinomas. As *AIPvar* have been rarely reported in prolactinomas occurring in FIPA kindreds, sporadic or resistant cases, in the current study we wanted to further characterize *AIPvar* associated prolactinomas as compared to relevant controls. To this end, we chose two study groups: 1) an unselected population of prolactinoma patients which represented a real-world population typical of those encountered in specialist (hospital) endocrine practice, in whom genetic testing is not routinely employed; 2) a population of DA-resistant prolactinomas with negative genetic testing.

From an epidemiological point of view, *AIPvar* prolactinomas had a median age at symptom onset and diagnosis that was >10 years younger than unselected and DA-resistant control; 75% were males. In addition, *AIPvar-*associated prolactinomas presented as large macroadenomas (median diameter 33.0 mm), with suprasellar extension, and frequent chiasmal compression and invasion. They differed from the unselected control group that was comprised predominantly (approx. 90%) of female microprolactinoma patients with a median age of 35 at diagnosis. The unselected control group responded well overall to DA treatment, with 82% having normal prolactin at a weekly cabergoline dose of ≤2.0 mg. As most of these patients had small microprolactinomas, it was not readily feasible to capture data on our shrinkage criteria (>50% from baseline) on MRI across this group. There were a few unselected prolactinoma patients that were either uncontrolled on DA or that had surgery (n=8), which reflects a hospital-based population (not community managed) and emphasizes that DA-led management is usually successful. With the other control group, we defined a population in which none of the known genetic causes of pituitary adenomas, including *MEN1* and *AIPvar* was present. That comparison shows that *AIPvar*-related prolactinomas have certain differences from DA-resistant tumors, and these characteristics might be relevant when considering which prolactinoma patients for genetic testing. Young males with large or giant, invasive prolactinomas were typical of the *AIPvar* group. In contrast, sex distribution (predominantly male), percentage of macroadenomas, and higher prolactin secretion at diagnosis did not discriminate *AIPvar*-related tumors from genetically-negative, DA-resistant controls. Indeed, unlike the DA-resistant group, in which a sub-set of patients are characterized by very high prolactin and large tumor size, in the *AIPvar* group prolactin levels increased little with rising tumor volume, which is unusual for prolactinomas in general (26). Furthermore, the *AIPvar* group had a lower rate of surgery, radiotherapy and a lower maximal cabergoline dose than the DA-resistant group. Control of hyperprolactinemia was achieved at the maximum DA dose in almost twice as many *AIPvar* patients as compared with the DA-resistant patients, indicating that while large and invasive prolactinomas are characteristic of *AIPvar*-related tumors, DA resistance is not absolute and control with DA or multimodal therapy is achievable. As noted by others, even large, complex prolactinomas related to *AIPvar* can have clinically-relevant hormonal and tumoral responses to chronic DA therapy (27). Unlike in the *AIPvar* group, the DA-resistant group had a sub-set of patients that are characterized by very high prolactin and large tumor size. The molecular pathways that explain why large prolactinomas can differ in terms of their secretory abilities remain unclear. Differing therapeutic responses to DA may be related to dopamine D2 receptor levels regulation, although this remains to be studied in *AIPvar*-related prolactinomas.

Overall, these results suggest that the main consequence of genetic disturbance of *AIP* favors earlier and more aggressive tumoral growth in prolactinomas. This echoes some, but not all, aspects of the more typical clinical presentation of *AIPvar-*related pituitary tumors, namely acromegaly and pituitary gigantism. In patients with *AIPvar-*related acromegaly, an earlier age at onset/diagnosis, male predominance, larger tumor size and relative resistance to medical treatment was seen as compared to *AIP* wild-type acromegaly controls (16). In *AIPvar-*related prolactinomas, we found additionally that invasion at baseline was a clinical characteristic that differentiated that group from DA-resistant and unselected subgroups. There was also a higher percentage of giant adenomas in the *AIPvar* group as compared with the DA-resistant group, which presumably contributed to higher rates of chiasmal compression in the *AIPvar* group. We did not observe cases of clinical or radiological apoplexy in any of the treatment groups in the study.

Our results suggest that when considering prolactinoma patients for *AIP* testing, DA-resistance should not be the only factor and should be accompanied by large and invasive tumoral growth in young patients. This echoes the negative results with using isolated somatostatin analog resistance as a factor for *AIP* testing in otherwise unselected acromegaly populations (28). Aggressive growth beginning at a young age was also a defining characteristic of *AIPvar*-related prolactinomas in other studies. In 90 pediatric patients prolactinomas, Kumar et al. reported on 18 cases of giant prolactinomas; of these, 18.8% of patients tested had an *AIP* pathogenic variant and *AIP* status was a predictor of requirement for other therapies in addition to DA (29). In a large study of 77 patients aged <20 with macroprolactinomas, Salenave et al. found that 5/55 (9%) of subjects had an *AIP* pathogenic variant, and 3/59 (5%) had a *MEN1* pathogenic variant. Interestingly, there was a relationship between DA-resistance and *MEN1* variant status, but not *AIPvar* status (30).

Numerous screening studies in series of patients with unselected pituitary adenomas, pediatric-onset pituitary adenomas, pituitary macroadenomas, and FIPA have been performed (13, 15, 17, 28, 30–42). About two-thirds of individuals with *AIPvar-*related pituitary adenomas present with acromegaly, usually in the setting of FIPA, and among this group, pituitary gigantism occurs in 32% (16). Prolactinomas are the second most common presentation (14.5%) and tumors with mixed growth hormone and prolactin secretion also occur (9.5%) (14). Among *AIPvar* patients, Hernandez Ramirez et al. reported that 7/31 variants occurred in patients with prolactinomas and of a total of 175 *AIPvar* positive subjects in the UK series, 19 (10.9%) had prolactinomas (27, 43). Although prolactinomas form an integral part of the *AIPvar* related spectrum of pituitary adenomas, they usually occur along with acromegaly in heterogeneous FIPA kindreds. In contrast, prolactinoma-only families that are a frequent presentation of FIPA are almost always *AIPvar* negative (14, 44). To date, only one FIPA family with homogeneous prolactinoma and a pathogenic *AIPvar* has been reported (27). In that Scottish family with a p.Arg304Ter pathogenic variant, there were four subjects with prolactinomas. The presentation ranged from a male in his 40’s with a giant partially cystic adenoma, to his sisters in their mid-30’s with microadenomas and amenorrhea (one sister had two pituitary lesions) and the son of one of the sisters with an asymptomatic but biologically active macroprolactinoma who was diagnosed on family *AIP* screening. Responses to cabergoline in that family were relatively good, although doses were limited by tolerability or were being up-titrated in three of the four reported patients.

How to adequately define DA resistance in terms of dose and response criteria remains unclear and has been the subject of significant debate (5). For this and previous work, we used the dose definition proposed by Molitch, which relates to the upper limit of the package insert/labelled dose of cabergoline, at 2.0 mg per week (18, 45). This cut-off has a logical basis, being derived from the highest dose at which safety and efficacy was established for hyperprolactinemia treatment from registration studies. As noted by Maiter, all cut-off dose levels of cabergoline or other DAs are essentially arbitrary, given that the ideal and safe dose to which patients can be up-titrated is unknown and depends on the strictness of the criteria for an acceptable hormonal and symptomatic response (5). Cabergoline is generally safe within its prescribed dose range and the cumulative risk of adverse events like cardiac valvulopathy has been shown to be low (46–48). Tolerability of DA treatment and its relationship with psychological disorders should always be assessed on a patient-by-patient basis (49, 50). The degree of shrinkage that is deemed clinically relevant is also arbitrary and a wide range of cut-offs have been suggested from 30-80% (5). The pragmatic approach is to be guided by the clinical status of the patient; for tumor shrinkage, the main goal should be alleviation of potential mass effects like optic chiasmal impingement. In the case of the mildly hyperprolactinemic patient on DA with sign/symptom resolution and whose tumor size is controlled, it is unclear if there is a tangible medical benefit to be gained by further up-titrating DA to achieve strictly numerically normal prolactin levels.

This study is a retrospective series in which the patient population was referred for genetic testing due to factors relating to an aggressive disease profile, including FIPA, prolactinomas occurring at a young age and an aggressive disease history. For this reason, we included a DA resistant control group that was genetically negative, and this allowed us to demonstrate that *AIPvar*-related prolactinomas have specific characteristics. The other unselected control group came from a single center, whereas the *AIPvar* and DA-resistant populations were multi-center, international cohorts. Local management decisions in the unselected controls could influence the comparisons, but in general therapeutic choices followed standard guidelines, so any center effect would be marginal. The comparisons on tumor shrinkage on DA were limited by the high frequency of very small micro-prolactinomas in the unselected controls. In the *AIPvar* group, the genetic variants seen were rare, but there remains uncertainty about their definitive pathogenic nature. This is an ongoing limitation in the field of *AIP* testing, as the functional data from *in vitro* models give varying interpretations of pathogenicity (25, 51–53). Altering testing recommendations based on this rare *AIP-*related presentation of prolactinomas should be done with caution. Like in acromegaly, there is no apparent value in performing *AIP* (or other) germline genetic testing in unselected prolactinomas at this time.

In conclusion, we confirm that prolactinomas occurring in sporadic and FIPA patients with germline *AIPvar* have aggressive clinical characteristics. In general, *AIPvar* confer a growth and aggression phenotype in prolactinomas, with larger, invasive tumors occurring at a younger age than in controls, but extremely elevated prolactin levels are not characteristic. These features differentiate *AIPvar* related prolactinomas from DA-resistant, genetically negative tumors. *AIPvar* and DA-resistant, genetically-negative groups were similar in terms of sex (predominantly male), and prolactin secretion at diagnosis. *AIPvar* related tumors were, however, not more challenging to treat than the wild-type DA resistant prolactinoma group, with the latter undergoing more frequent surgery, radiotherapy and using a higher maximal cabergoline dose.

## Data availability statement

The raw data supporting the conclusions of this article will be made available by the authors, without undue reservation.

## Ethics statement

The studies involving human participants were reviewed and approved by Hospitalo-Facultaire Ethics Committee, University of Liege. The patients/participants provided their written informed consent to participate in this study.

## Author contributions

LV, ABe and AD conceived of and designed the study. LV, SC, TC, M-LJ-R, MC, LN, AF, AE, AK, TB, ABa and AD identified subjects and collected clinical information on active and control groups. PB and PR conducted genetic analyses. LV, AD, PP designed the study database. PP conducted statistical analyses. LV, PP and AD wrote the first draft of the manuscript. All authors contributed to the article and approved the submitted version.
